# Supplementary material for: Glycolytic Reprogramming in Silica-Induced Lung Macrophages and Silicosis Reversed by Ac-SDKP Treatment
Source: Int J Mol Sci. 2021 Sep 17;22(18):10063. doi: 10.3390/ijms221810063 (PMC8465686; doi:10.3390/ijms221810063)
Supplement: Supplementary file 1 [file ijms-22-10063-s001.zip › ijms-1372648-supplement.pdf]

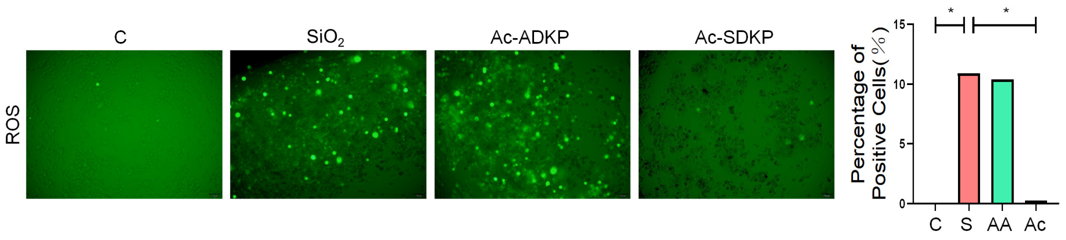

Figure S1. Effects of Ac-SDKP on the intracellular ROS production in macrophages treated with silica by using DCFH-DA staining, bar = 100 μm. *Compared with corresponding control group, *P* < 0.05.

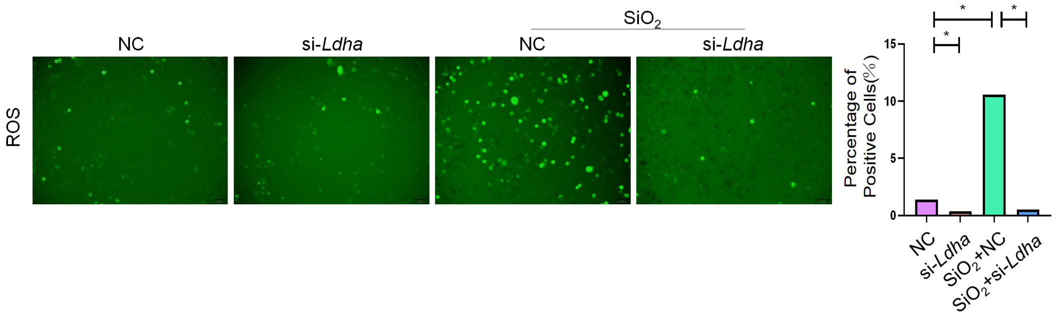

Figure S2. Effect of LDHA-siRNA on the intracellular ROS production in macrophages treated with silica by using DCFH-DA staining, bar = 100 μm. *Compared with corresponding control group, *P* < 0.05.
